# Non-equilibrium approach for binding free energies in cyclodextrins in SAMPL7: force fields and software

**DOI:** 10.1007/s10822-020-00359-1

**Published:** 2020-11-24

**Authors:** Yuriy Khalak, Gary Tresadern, Bert L. de Groot, Vytautas Gapsys

**Affiliations:** 1 Computational Biomolecular Dynamics Group, Department of Theoretical and Computational Biophysics, Max Planck Institute for Biophysical Chemistry, 37077 Göttingen, Germany; 2 Computational Chemistry, Janssen Research & Development, Janssen Pharmaceutica N. V., Turnhoutseweg 30, 2340 Beerse, Belgium

**Keywords:** Alchemy, Non-equilibrium free energy calculations, Force field

## Abstract

In the current work we report on our participation in the SAMPL7 challenge calculating absolute free energies of the host–guest systems, where 2 guest molecules were probed against 9 hosts-cyclodextrin and its derivatives. Our submission was based on the non-equilibrium free energy calculation protocol utilizing an averaged consensus result from two force fields (GAFF and CGenFF). The submitted prediction achieved accuracy of ${1.38}\,\hbox {kcal}/\hbox {mol}$ in terms of the unsigned error averaged over the whole dataset. Subsequently, we further report on the underlying reasons for discrepancies between our calculations and another submission to the SAMPL7 challenge which employed a similar methodology, but disparate ligand and water force fields. As a result we have uncovered a number of issues in the dihedral parameter definition of the GAFF 2 force field. In addition, we identified particular cases in the molecular topologies where different software packages had a different interpretation of the same force field. This latter observation might be of particular relevance for systematic comparisons of molecular simulation software packages. The aforementioned factors have an influence on the final free energy estimates and need to be considered when performing alchemical calculations.

**Electronic supplementary material:**

The online version of this article (10.1007/s10822-020-00359-1) contains supplementary material, which is available to authorized users.

## Introduction

The computational chemistry community benefits greatly from the periodically organized blinded challenges providing an unbiased evaluation of the state-of-the art techniques available in the field. Over the years the SAMPL challenge has provided opportunities for scientists to predict ligand solvation free energies, octanol-water partition coefficients, protein-ligand and host–guest binding free energies [1–6].

The previous SAMPL challenge (SAMPL6) [5] contained an additional SAMPLing sub-challenge [7], where we took part by probing the sampling efficiency of the non-equilibrium alchemical free energy calculation approach for the absolute binding free energies of host–guest systems. Previously, we have also shown the potential of the non-equilibrium alchemical method in a post-submission evaluation of a dataset from the D3R Grand Challenge 4 by calculating relative binding free energies for a protein-ligand complex [8]. A similar approach employing non-equilibrium uni-directional transitions has also been applied in SAMPL6 by Piero Procacci’s group for calculating water-octanol partition coefficients [9] and host–guest binding free energies [10].

**Fig. 1:**
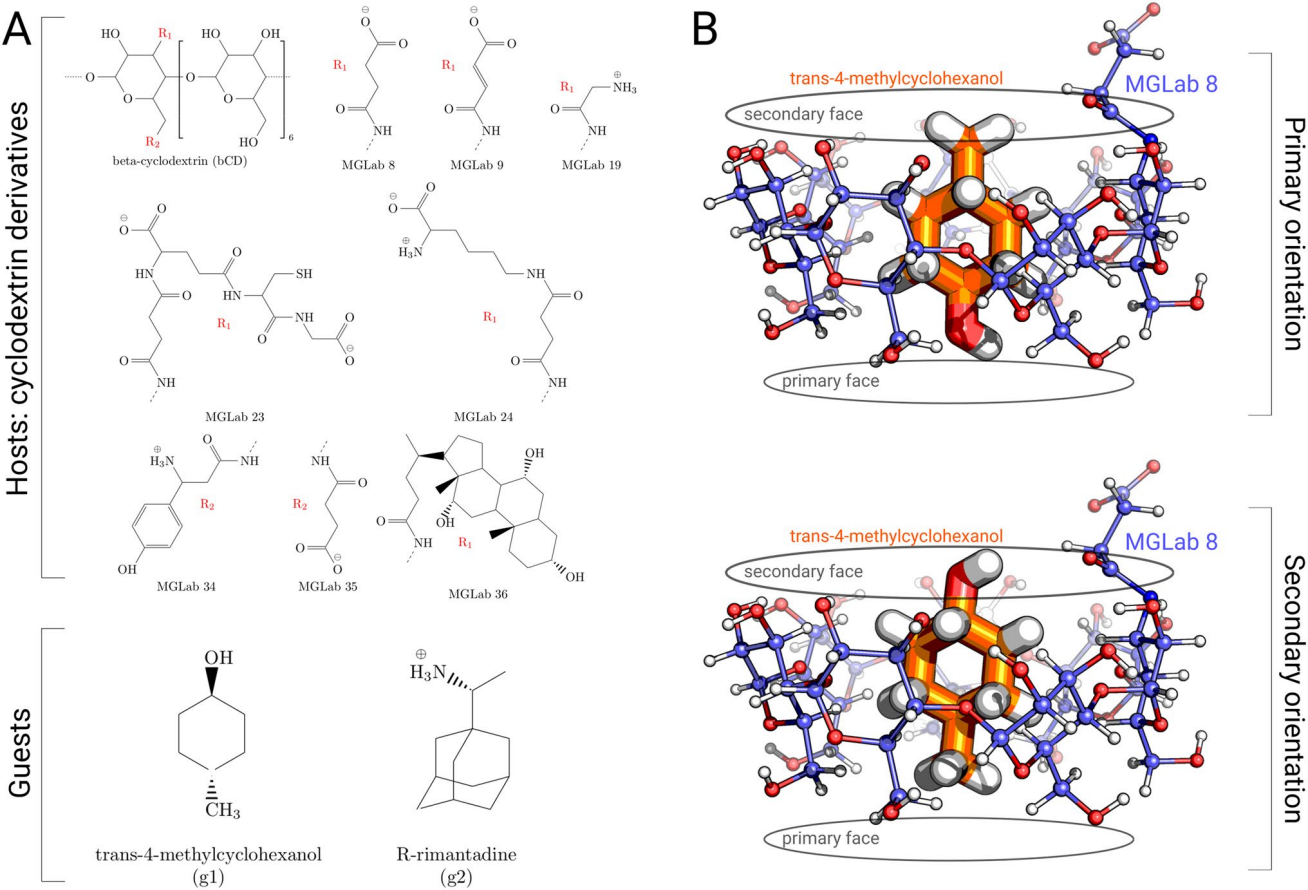
Summary of the cyclodextrin derivative category of the SAMPL7 challenge. The free energy calculations were performed for 9 hosts binding 2 guests (**a**). Those $\text {R}_x$ substituents that are not shown explicitly correspond to hydroxy groups. In the simulations we probed two guest orientations: primary orientation (hydroxy group of g1 and amine of g2 oriented towards the primary face) and secondary orientation (hydroxy group of g1 and amine of g2 oriented towards the secondary face) (**b**)

Considering these successful applications of the non-equilibrium approaches in the previous challenges, we took part in the blind prediction of the host–guest binding free energies in the framework of SAMPL7. Of the three systems offered for investigation, we concentrated on the analysis of 9 cyclodextrin derivatives binding to 2 guest molecules, trans-4-methylcyclohexanol and rimantadine (Fig. 1a).

Our ranked submission for the challenge contained a calculation based on the non-equilibrium free energy obtained using an averaged consensus result from two force fields: GAFF [11] and CGenFF [12]. In the SAMPL7 challenge, the cyclodextrin category contained only two ranked submissions: Procacci et al. and our calculations, both relying on similar methodology, but different ligand and water force fields. The two submissions showed a small difference in accuracy with the revealed experimental measurements: in terms of average unsigned error (AUE) $1.01\pm {0.17}\,\hbox {kcal}/\hbox {mol}$ for Procacci et al. and $1.38\pm {0.06}\,\hbox {kcal}/\hbox {mol}$ for our calculation, and in terms of Pearson correlation 0.19 ± 0.17 and 0.18 ± 0.06, respectively.

In the current report we firstly describe in detail our calculation setup and provide deeper analysis of the individual force field performance. Subsequently, we investigate the underlying reasons for the discrepancies between the two submissions that utilize similar methodological approaches, yet different versions of the GAFF force fields and water models.

## Methods

### Initial simulations

In the first part of this study we calculate binding free energies of trans-4-methylcyclohexanol (g1) and R-rimantadine (g2) to a series of cyclodextrin derrivatives (Fig. 1a) using the GAFF 1.81 [11, 13] and CGenFF 4.1 [12, 14] force fields. The average of the free energies obtained with both of these force fields represents our consensus approach [15–18] and serves as our ranked submission to the SAMPL7 challenge.

For GAFF 1.81, AM1-BCC charges [19] were derived with AmberTools 19 and ACPYPE [20] was used to convert the parameters to a Gromacs compatible format. CGenFF parameters were obtained using the https://cgenff.umaryland.edu webserver [21]. Ionization states from the latest version of the SAMPL7 challenge’s GitHub repository were used. Initial structures were generated by positioning the guest so its center of geometry equals to that of the host while the amine (for R-rimantadine) and hydroxyl (for trans-4-methylcyclohexanol) were pointing to the primary or secondary cyclodextrin face [22]. In the calculation we explicitly probed two orientations, corresponding to two possible binding poses, for each host–guest system (Fig. 1b). Binding in the primary orientation occurs when the polar group of the guest is pointing to the primary face of the host (the face with only one hydroxyl group per sugar residue). Conversely, in the secondary orientation the polar group of the guest is pointing to the secondary face of the host (two hydroxyls per residue). This is the orientation preferred by rimantadine (g2) in native beta-cyclodextrin [23].

Separate sets of simulations were carried out for each of these binding poses for each host–guest system. The reported absolute free energies were computed by taking a Boltzmann average of contributions from both available poses [24]. The events of orientation flipping or ligand unbinding were filtered out during the analysis.

As the major population of g2 under neutral pH is charged, the double-system/single-box method was used to keep the net charge of the system constant during the non-equilibrium simulations [25]. Simulation boxes were set up by adding a second guest molecule at a distance of ${3}\,\hbox {nm}$ from the host and adding ${1.5}\,\hbox {nm}$ of padding between the resulting solute and the box edges. In one end state of the alchemical transition, the second guest was coupled to the system, while the guest bound to the host was decoupled and vice versa for the other end state. Harmonic position restraints with a force constant of ${1000}\,\hbox {kJ}\,\hbox {mol}^{-1} \hbox{nm}^{-2}$ were applied to one atom of the host and one atom of the second guest to keep them beyond cutoffs of each other. The system was solvated with TIP3P water [26]. Sodium and chloride ions were introduced to neutralize the system and reach a ${25}\,\hbox {mM}$ salt concentration. No ions were placed within ${0.3}\,\hbox {nm}$ of the solute. In the case of simulations with GAFF force field, ion parameters of Joung and Cheatham [27] were used, while for CGenFF simulations original chloride parameters of Roux [28] were used in combination with newer sodium and sodium-chloride interaction parameters [29].

Simulations were performed with Gromacs 2019.4 [30] at a temperature of 300.15 K using the stochastic dynamics integrator with an inverse friction constant of ${2}\,\hbox {ps}$ and a time step of ${2}\,\hbox {fs}$. Van der Waals interaction cutoff of ${1.1}\,\hbox {nm}$ with a switching function starting at ${1.0}\,\hbox {nm}$ was employed. Particle mesh Ewald with the real space cutoff of 1.1 nm, interpolation order of 4 and Fourier spacing of 0.12 nm was used to treat electrostatic interactions [31, 32]. For each combination of host, guest, binding pose, and end state (first guest coupled and second decoupled and vice versa) six equilibrium trajectories were generated as described below. To retain the decoupled first guest in the relevant binding pose, harmonic relative restraints (force constants of ${4184}\,\hbox {kJ}\,\hbox{mol}^{-1} \hbox{nm}^{-2}$) for distance and (${41.84}\,\hbox {kJ}\,\hbox{mol}^{-1} \hbox{rad}^{-2}$) for angles and dihedrals were applied [33]. Additional position restraints with a force constant of ${9000}\,\hbox {kJ}\,\hbox{mol}^{-1} \hbox{nm}^{-2}$ for GAFF and ${1000}\,\hbox {kJ}\,\hbox{mol}^{-1} \hbox{nm}^{-2}$ for CHARMM were introduced for all solute atoms for equilibration. Following initial energy minimization, a ${300}\,\hbox {ps}$ NVT simulation was performed. The additional position restraints were then disabled and a ${20}\,\hbox {ns}$ production simulation was carried out in the NPT ensemble with the Parrinello-Rahman barostat [34, 35] using a ${5}\,\hbox {ps}$ time constant. The first ${5}\,\hbox {ns}$ of this simulation were discarded as final equilibration.

For each combination of force field, host, guest, binding pose, and end state three repeats where the first guest did not unbind or flip to the opposite binding pose were chosen for thermodynamic integration to the opposite end state. Binding poses where this was not possible were discarded as too weakly binding to significantly contribute to the overall absolute free energy of binding. For each such repeat 151 initial frames separated by ${50}\,\hbox {ps}$ were extracted from the equilibrium trajectories and ${500}\,\hbox {ps}$ NPT simulations were run from each one driving the system to the opposite end state by linearly changing lambda.

The free energy difference was estimated using a maximum likelihood estimator [36] based on the Crooks Fluctuation Theorem [37] as implemented in the pmx package [38]. For the final free energy estimate an analytical correction [33] due to the effect of the relative restraints was added. The value for each binding pose was taken as the average of the three independent simulation repeats. Finally, the average value over the two force fields is reported as our estimate for the absolute free energy of binding. The uncertainties for free energies were calculated as standard errors of the mean when considering independent simulation repeats.

After the reference experimental data and all the submissions for the SAMPL7 challenge were released, we performed additional calculations including GAFF 1.81, GAFF 2.1, GAFF 2.11 and modified versions of these force fields, as well as two water models (TIP3P and OPC3 [39]).

### Further investigation of GAFF2

To elucidate the reason for the systematic shift in the calculated free energies observed for GAFF 2.11 Fig. 4 and the disagreement with the GAFF 2.1 results reported by Procacci et al., a series of free energy estimates using a set of modified GAFF 2.1 versions were carried out. For this investigation, a slightly adjusted version of the calculation protocol was employed. The three simulation repeats shared the same initial equilibration in the NVT ensemble which was increased to ${0.5}\,\hbox {ns}$. Also, an additional equilibration step was added, heating the system from 0 to 298 K during 0.5 ns prior to the production run.

## Results

### Ranked submission

**Fig. 2:**
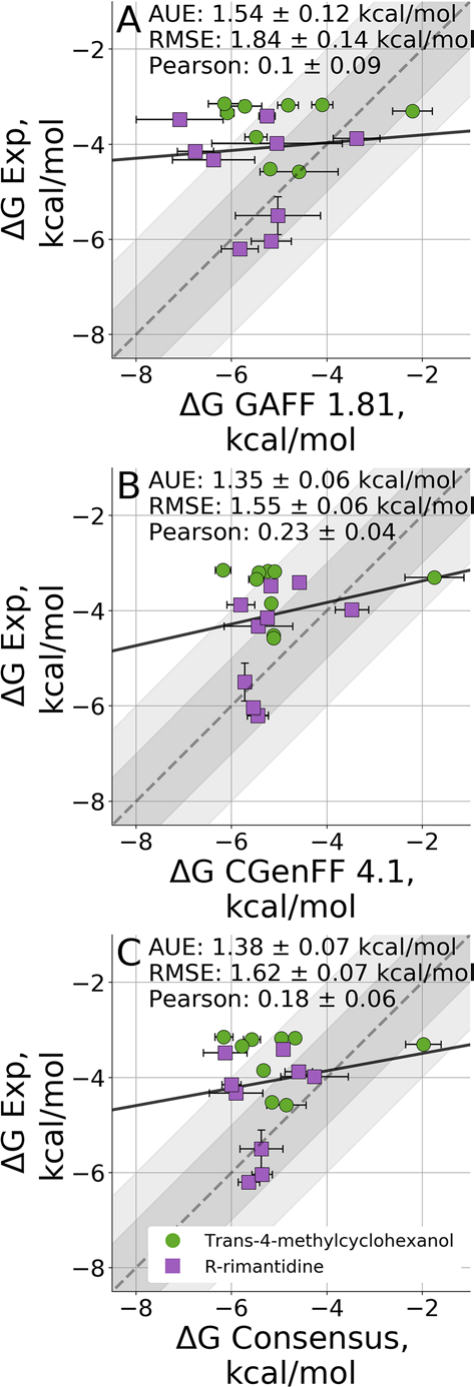
Absolute binding free energies with GAFF 1.81 (**a**) and CGenFF 4.1 (**b**) force fields as well as the consensus average of the two (**c**). The grey and light grey regions represent 1 and ${2}\,\hbox {kcal}/\hbox {mol}$ deviations from experiment respectively, while the solid line is a linear fit through the data points

From previous experience with relative ligand binding free energies [8, 15, 18] we have seen that using a consensus approach averaging over the predictions of multiple force fields can help reduce bias induced by the parametrization of any individual force field. Therefore, we used a consensus of GAFF 1.81 and CGenFF 4.1 as our ranked submission to the SAMPL7 challenge (Fig. 2).

For rimantadine the primary orientation proved to be unstable in CGenFF 4.1, flipping to the secondary orientation in most equilibrium simulations. Meanwhile, in GAFF 1.81 the primary orientation was unstable only for MGlab 8 with g1. GAFF 1.81 also significantly overestimates the binding affinity to several cyclodextrin hosts. Binding to MGLab 23, 24 and 36 is overestimated by more than ${2}\,\hbox {kcal}/\hbox {mol}$ for both guests. Concerned with the possibility of insufficient sampling of the degrees of freedom of the side chains, we extended both the equilibrium and non-equilibrium sampling for these hosts as well as for MGLab 19. For each extended simulation we performed 3 independent simulations of 200 ns equilibrium sampling followed by 651 transitions of 0.5 ns each for both forward and reverse directions. However, while we were able to achieve better convergence the prediction accuracy remained unchanged (Fig. S1).

CGenFF 4.1 exhibits a similar, albeit smaller, overestimation for these same hosts with long side chains (MGLab 23, 24 and 36), indicating that the problem to some extent is shared between the two force fields.

The consensus force field approach in the current application performed comparably to the better performing CGenFF force field. Averaging the GAFF and CGenFF results mitigated the worst overbinding predicted by GAFF 1.81 as well as the underestimation of free energy for the MGLab 8-g1 outlier in CGenFF 4.1. Even though the consensus method did not provide an additional improvement in accuracy, it allowed for a reliable way to combine the results from two force fields, where otherwise an arbitrary choice for the final submission would have had to be made.

### Learning from force field differences

The observed differences (and consistencies) among the force field variants may suggest deeper insight into the underlying reasons for the prediction accuracy. Here, we had a closer look into one of the major outliers: MGLab 24 host binding to R-rimantidine. The host MGLab 24 contains a long sidechain (Fig. 1) and shows lower than average binding affinity to rimantidine across the examined set of ligands $({-4.15}\,\hbox {kcal}/\hbox {mol}$). Interestingly, another host MGLab 9 binds to rimantadine with a similarly low affinity of ${-3.88}\,\hbox {kcal}/\hbox {mol}$, yet its sidechain is much smaller. This observation suggests that the sidechain size may not necessarily correlate with the binding affinity.

Predictions made with the CGenFF force field in part match this experimental observation: binding affinities for R-rimantidine and both guests, MGLab 24 and MGLab 9, are comparable $(-5.25 \pm {0.11}\,\hbox {kcal}/\hbox {mol}$ and $-5.81 \pm {0.29}\,\hbox {kcal}/\hbox {mol}$). While there is an overall offset in the calculated free energies, both hosts are estimated to interact with the guest with a similar affinity. In contrast, GAFF 1.81 predicts very different binding affinities for R-rimantidine with MGLab 24 and MGLab 9: $-6.76 \pm {0.38}\,\hbox {kcal}/\hbox {mol}$ and $-3.38 \pm {0.49}\,\hbox {kcal}/\hbox {mol}$, respectively. This suggests a different interpretation of binding by CGenFF and GAFF force fields which manifests in a prediction of an overly strong binding affinity between R-rimantidine with MGLab 24 by GAFF.

**Fig. 3:**
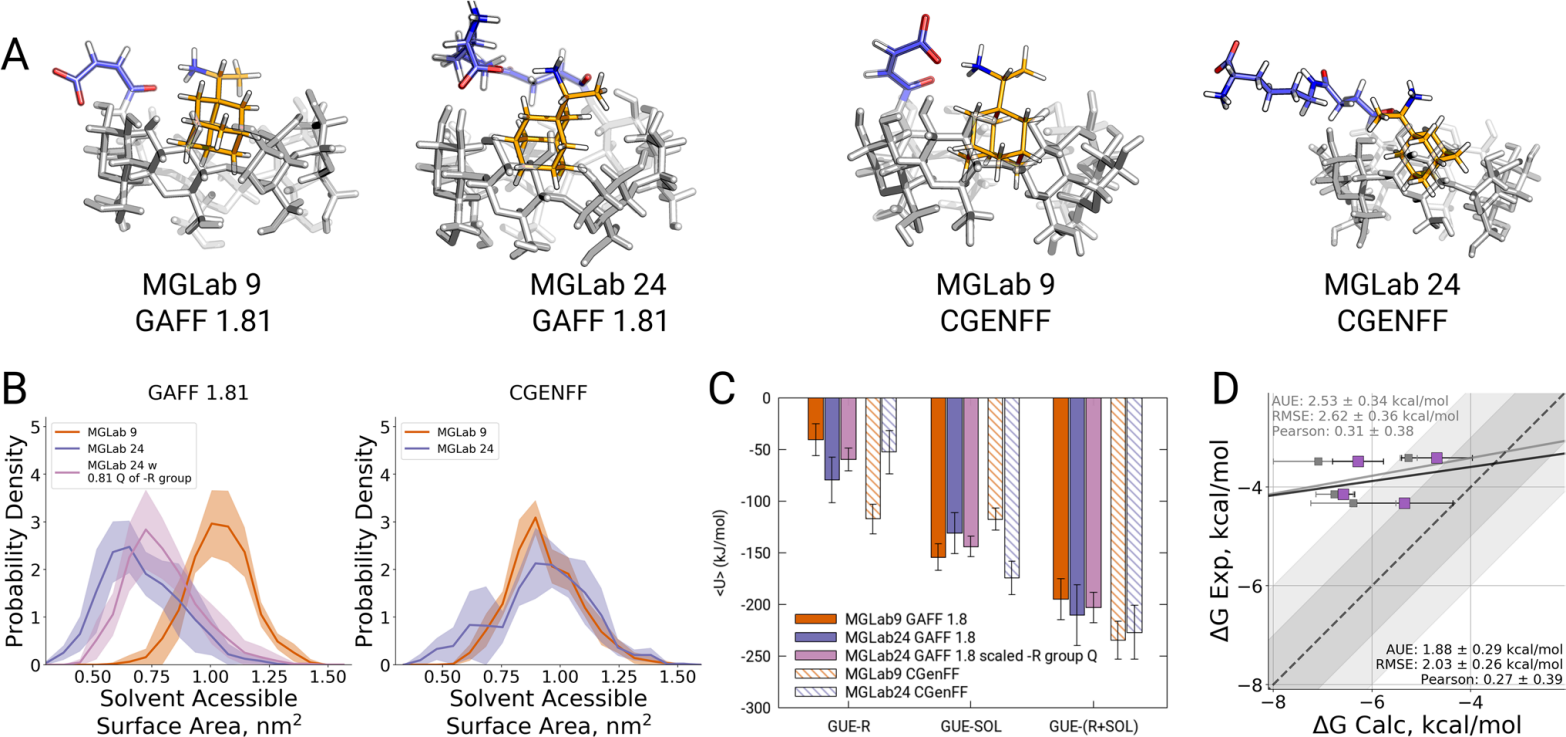
Representative structures for R-rimantidine bound in its secondary orientation to the hosts MGLab 24 and MGLab 9 when simulated with GAFF 1.81 and CGenFF force fields (**a**). Solvent accessible surface area of R-rimantidine when bound to a host in its secondary orientation (**b**). Interaction energies between R-rimantidine and the host sidechain (GUE-R), R-rimantidine and water (GUE-SOL) and the overall interaction energy between R-rimantidine and the sidechain and water (GUE-(R+SOL)) (**c**). The $\Delta$G values calculated for R-rimantidine binding to MGLab 19, 23, 24 and 36 with GAFF 1.81 force field after scaling the charges of the host sidechains (purple). $\Delta$G calculated with the original GAFF 1.81 topologies are shown in gray (**d**)

Comparison of the structural ensembles generated by the molecular dynamics simulations (representative structures in Fig. 3a) and solvent accessible surface area for the bound guest molecule (Fig. 3b) reveal clear differences between the force fields. The host sidechain in CGenFF is mostly solvated and has only limited interactions with the guest for both cases, MGLab 24 and MGLab 9. As a consequence, R-rimantidine remains largely exposed to solvent when bound to either host, thus exhibiting similar binding affinity to each of them. The simulations in GAFF force field show a different interaction: here, the long sidechain of MGLab 24 strongly interacts with the guest, reducing its solvent accessible surface area and altering binding affinity. This interplay between the guest-sidechain and guest-solvent interactions is also well reflected in the interaction energies calculated from the simulated ensembles (Fig. 3c).

Narrowing down the particular differences in the force fields that lead to such disparities is hardly feasible, as the description of molecular topologies for GAFF and CGenFF differ in multiple terms, including force constants, equilibrium bond, angle, dihedral values, non-bonded parameters and even functional form of the potential. In the current study, we limit the scope of force field comparison to the partial charges of the host side chains. Comparison of the topologies for the hosts showing largest outliers in terms of the calculated $\Delta$G revealed that the charges in GAFF are consistently larger than those in CGenFF (Fig. S2). To probe, how the calculation results would be affected by making the GAFF topologies more similar to those of CGenFF, we scaled down (factor of 0.81) the GAFF sidechain partial charges for the hosts MGLab 19, 24, 23 and 36.

This modification indeed had an expected outcome: the interactions between R-rimantidine and host sidechains were reduced, while sidechain-solvent interactions increased (Fig. 3c). Consequently, the guest became more exposed to the solvent (Fig. 3b). In turn, the calculated free energy differences for the GAFF topology with scaled charges are closer to the experimental values (Fig. 3d). This simple experiment illustrates that it is possible to rationalize the differences in the outcomes from different force fields and further exploit them to improve the prediction accuracy.

### Comparison to the other submission

After both the ranked submissions and the experimental results became public, we noted a methodologically similar submission by Procacci et al. Namely, a non-equilibrium approach based on a unidirectional (decoupling only) estimator, enhanced sampling of the end states, a harmonic restraint between the centers of mass for host and guest, and use of the GAFF 2 force field in combination with OPC3 [39] water model and the ORAC simulation engine [40].

**Fig. 4:**
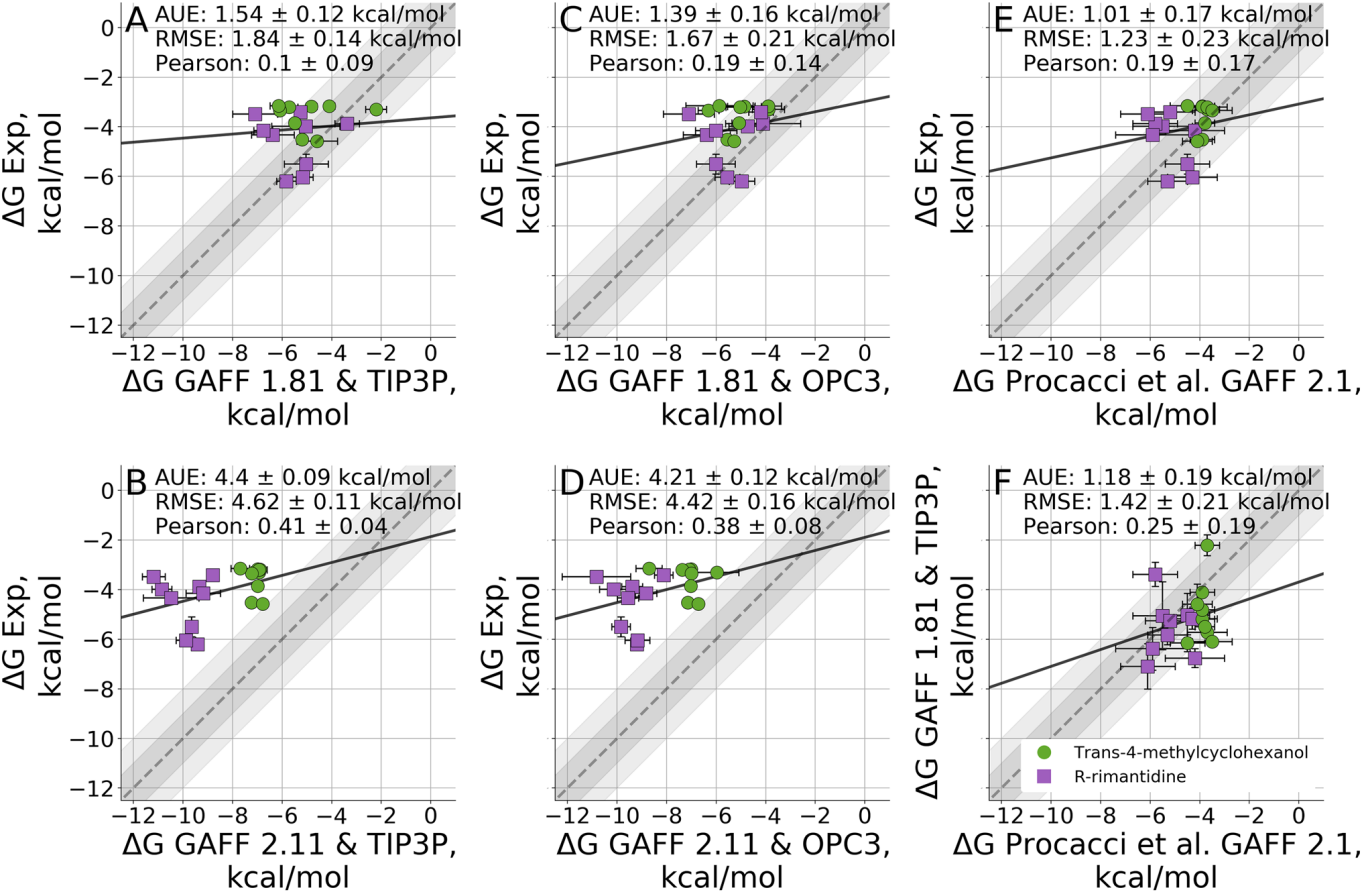
Comparison of absolute binding free energies with GAFF versions 1.81 (**a**, **c**) and 2.11 (**b**, **d**) using TIP3P (**a**, **b**) and OPC3 (**c**, **d**) as water models. The results submitted by Procacci et al are compared to the experimental values (**e**) and to our calculations with the GAFF 1.81 force field (**f**). The grey and light grey regions represent 1 and ${2}\,\hbox {kcal}/\hbox {mol}$ deviations from experiment respectively, while the solid line is a linear fit through the data points

For a more direct comparison of our approach and that of Procacci et al., we computed the binding free energies by probing both, GAFF 1.81 and GAFF 2.11 force fields, in combination with TIP3P, as well as OPC3 water (Fig. 4).

The results obtained with GAFF 1.81 are comparable to those of Procacci et al. with GAFF 2.1 (Fig. 4f) in terms of AUE $(1.18\pm {0.19}\,\hbox {kcal}/\hbox {mol})$, although the correlation is weak (0.25±0.2). Replacing the TIP3P water model with OPC3 had only a minor effect within the level of the estimated uncertainty for both force fields, GAFF 1.81 and GAFF 2.11. The overbinding of MGLab 23, 24 and 36 was present irrespective of the water model.

The more unexpected result was the overbinding observed for all hosts with GAFF 2.11 regardless of the water model. The majority of this effect comes from the secondary orientation, which was found to have consistently stronger binding free energies (Fig. S3) with the average free energy difference of ${1.5}\,\hbox {kcal}/\hbox {mol}$ between the two orientations and some values reaching ${4.8}\,\hbox {kcal}/\hbox {mol}$. Such strong overbinding was in stark disagreement with the values reported by Procacci et al.

### Inspecting GAFF 2.1 force field

The above disagreement between the force fields is peculiar, since partial charges have not been altered between the force fields and the Lennard–Jones parameters are comparable as well.

Reparameterizing the systems with GAFF 2.1 to match the choice of force field version of Procacci et al. yielded little change from GAFF 2.11 (Fig. S4). This was not surprising, as for the systems in question the two force field versions have only minor differences in their force constants for bond and angle potentials.

#### GAFF 2.1 sugar specific dihedrals

A comparison of the topologies for the GAFF 1.8 and GAFF 2.1 force fields generated by antechamber v17.3 from AmberTools 17, revealed major differences in the parameters of dihedral angles. A further inspection of the dihedral angle definitions in these force fields revealed that there exist multiple dihedral definitions in the GAFF 2.1 (as well as GAFF 2.11) force fields where an identical set of atom types is assigned different sets of dihedral parameters. These overdefined dihedrals are atom type specific (i.e. do not contain wildcard atom types), have the same multiplicities (i.e. several dihedrals with the same atom types and identical multiplicities) and are entered in the force field files non-sequentially (i.e. there are other dihedral definitions separating the overdefined entries). All this indicates that in some cases (see SI) the identical atom mappings may have been ambiguously assigned multiple sets of parameters.

In particular, several such problematic dihedral definitions were specifically designated to parameterize sugar molecules. For example, for the c3-c3-os-c3 dihedrals in the sugar rings of the hosts antechamber would assign the more general purpose dihedral set even though a set of parameters with higher force constants specific to sugars was available. We reported this finding to the GAFF 2 developers (personal communication).

**Fig. 5:**
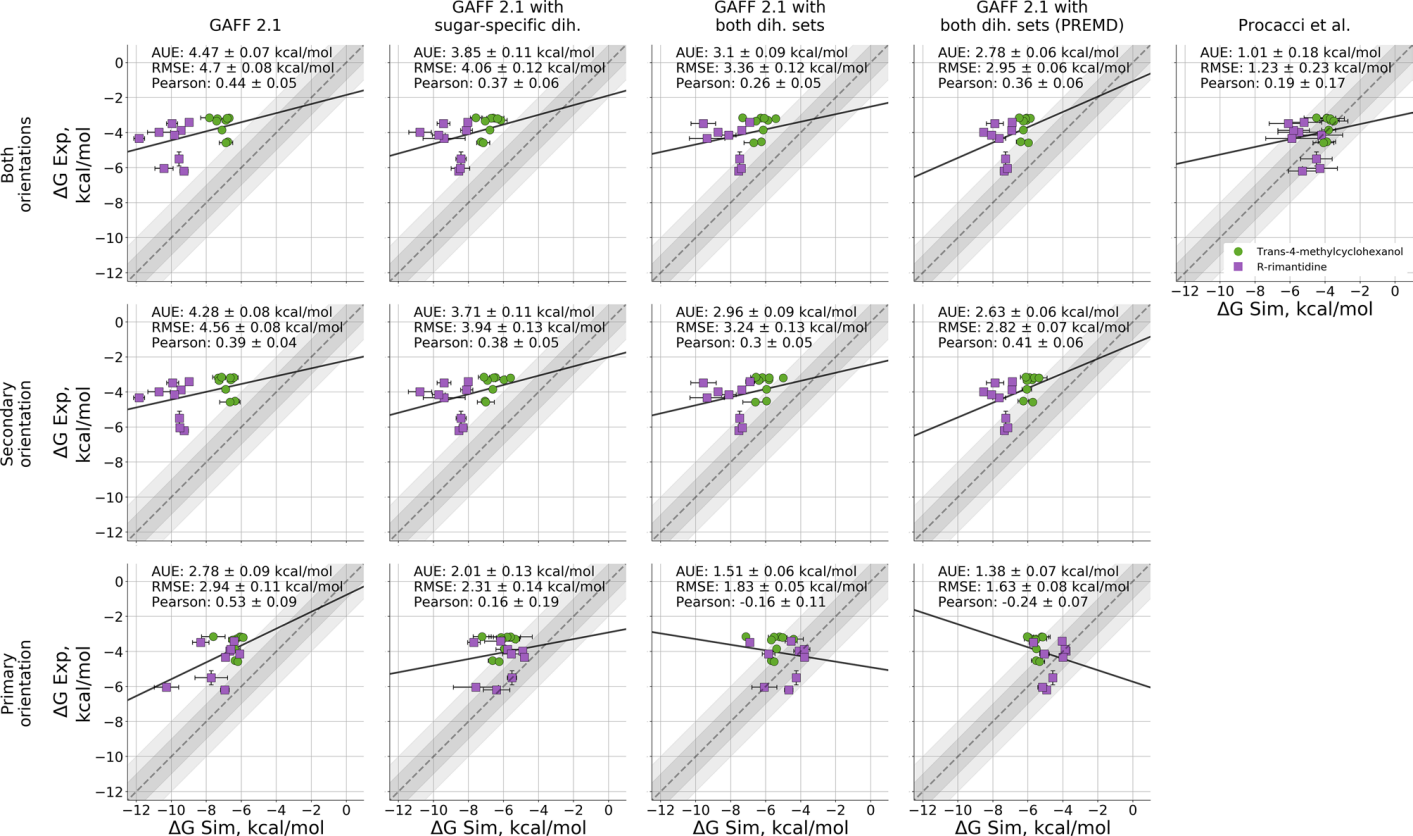
Comparison of absolute binding free energies obtained for different changes to the dihedral potentials of GAFF 2.1. Each step brings the potential closer to the one employed in ORAC

As a test, we have adjusted our topologies to solely use the sugar-specific dihedral parameters for the cyclodextrin derivatives. This resulted in a reduction of the predicted binding affinities, bringing them closer to the experimental measurements by more than ${0.5}\,\hbox {kcal}/\hbox {mol}$ in terms of AUE (Fig. 5b). This, however, was insufficient to reach the quality of agreement with experiment as obtained by GAFF 1.81.

#### Over-defining GAFF 2.1 dihedrals

A consultation with Procacci et al. and inspection of their topologies revealed that the GAFF 2.1 topologies generated by antechamber and PrimaDORAC [41] (software used by Procacci et al.) differ. One of the main disparities was the interpretation of the dihedral parameter assignment from the force field definition to the dihedral angles identified in the topology. PrimaDORAC used all specific (no wildcard atoms) dihedral potentials found in the force field, thus overdefining a dihedral multiple times for the case of GAFF 2.1. This way, certain dihedrals in the topologies for host molecules contained multiple parameter sets at once, in turn defining a different potential energy landscape to be sampled by the molecular dynamics engine.

We probed the effect of overdefining the sugar specific dihedral sets as done by PrimaDORAC (Fig. 5c). This, in turn further reduced the binding affinities and brought the estimates closer to the experimental measurement. The offset in binding free energies was reduced in both primary and secondary binding orientations, although for the secondary orientation, the overbinding effect still remained. The overall offset in the calculated free energies also remained, AUE of ${3.1}\,\hbox {kcal}/\hbox {mol}$.

#### Enhanced sampling

It is plausible that the free energy surface for the two force field versions, GAFF 1.8 and GAFF 2.1, may have different barrier heights. Thus, it cannot be excluded that when switching the force field version we have encountered an under sampling issue. To ensure that our predictions are not suffering from under sampling, we recomputed the free energies with the modified GAFF 2.1 force field version employing enhanced sampling via partial replica exchange molecular dynamics (PREMD) [42]. This approach is similar to the replica exchange with solute tempering (REST) method [43], albeit PREMD requires an additional assumption that the coupling between the regions coupled to the separate thermostats is weak. To have the dihedral angle definitions as similar to those generated by PrimaDORAC we replaced as many dihedrals as possible from our topology with those from the topology shared with us by Piero Procacci (personal communication).

The simulations were performed by using 8 temperature replicas ranging from 298 to 928 K. Only the host molecule was coupled with thermostats of higher temperature. A weak harmonic potential of ${10.40}\,\hbox {kJ}\,\hbox {mol}^{-1} \hbox{nm}^{-2}$ was used to restrain the centers of mass of the guest molecule in complex with host. This setup brings us to an approach closer to that used by Procacci et al. The resulting free energy accuracy, however, improves only marginally (Fig. 5d). This indicates that under sampling is not a likely explanation to the observed shift in the free energy estimates.

Modifications of the dihedral angle parameters and further sampling enhancement have reduced the estimated binding affinities. In terms of absolute agreement with the experimental measurement, binding in the primary orientation has reached AUE of ${1.38}\,\hbox {kcal}/\hbox {mol}$ after overdefining the dihedrals and applying PREMD. However, binding in the secondary orientation still dominates the overall binding affinity, as it exhibits higher affinity between the host and the guest. This is in contrast to the observation from calculations with the GAFF 1.81 force field, where both binding poses contributed comparably to the overall binding (Fig. S5). Procacci et al. report having probed both binding orientations for some of the host–guest pairs, finding only neglibible contribution from the primary orientation. These observations suggest that there still exists a marked difference between the adjusted GAFF 2.1 and GAFF 1.81 force fields, as well as between our calculations and those of Procacci et al.

Upon further inspection we identified other discrepancies between the antechamber and PrimaDORAC topologies for the GAFF 2.1 force field. Namely, the atom type assignment is not identical between the two software packages, resulting in further differences in bond, angle and dihedral parameters. It is clear that the overbinding can be reduced by fully reverting the topologies to the GAFF 1.81 version as shown in Fig. 2a or using GAFF 2.1 PrimaDORAC version as demonstrated by Procacci et al. Fig. 5e. These observations might be helpful for the force field developers to further narrow down the problematic atom type and/or force field parameter assignments.

### Structural analysis

**Fig. 6:**
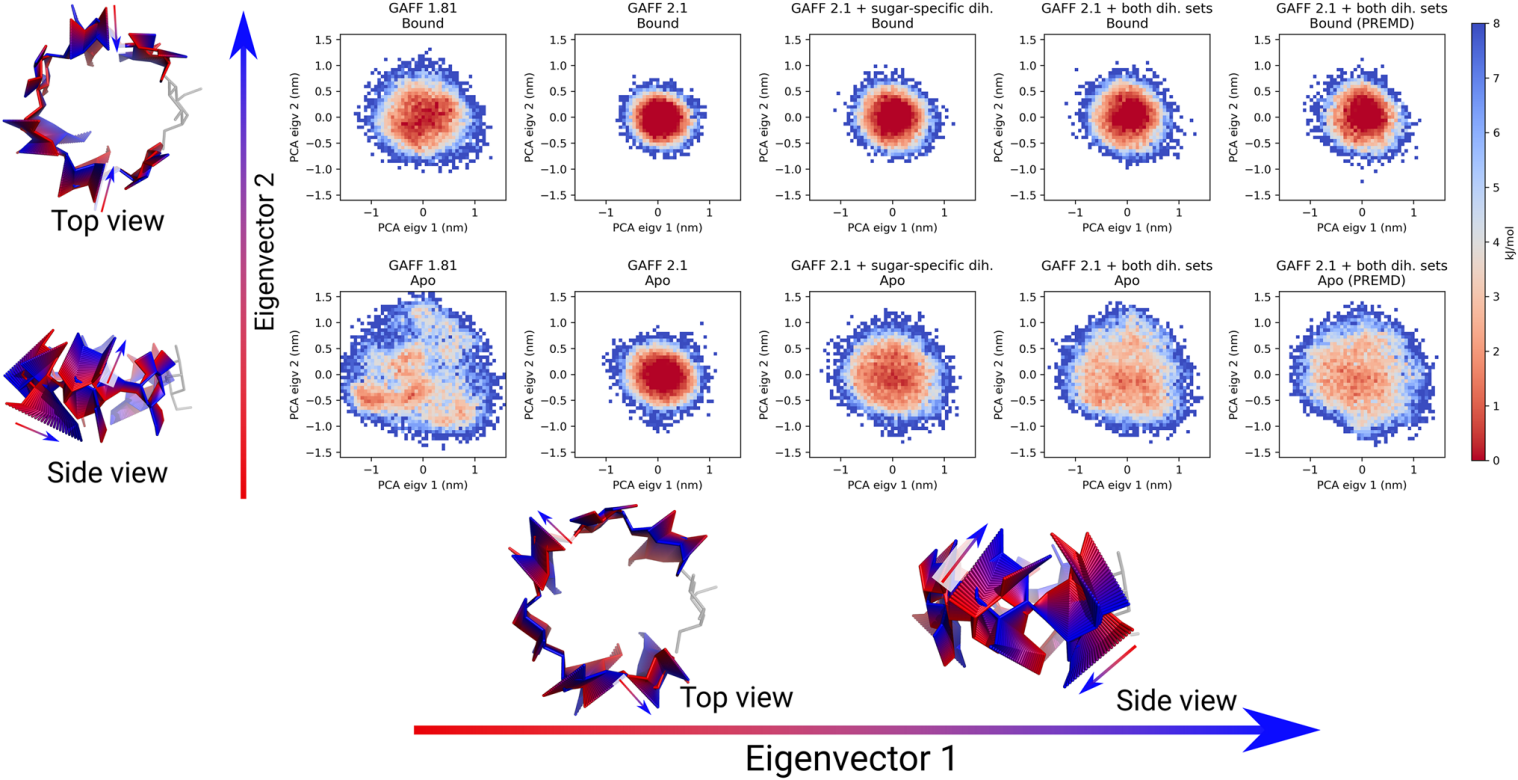
Free energy surfaces of all host conformations obtained with different force field variants. The principal components were calculated by combining apo and holo trajectories from GAFF 1.81 force field. All simulations depicted use TIP3P water and the PCA analysis was carried out using only non-modified sugars of the hosts. States corresponding to surface binding have been filtered out. Bound states (top row) explore less space than unbound ones (bottom row). Structural representations depict interpolations along the two principal components with the largest eigenvalues

To understand the underlying structural reasons for the overly strong binding in the GAFF 2 force field we have projected the host trajectories on the two principal components with the largest eigenvalues (Fig. 6). Trajectories where the guests are not bound to the hosts explore a much wider range of configurations than the bound ones. Meanwhile, the projections show that GAFF 1.81 trajectories are much less confined than GAFF 2.1 trajectories. Similar behavior has been previously observed by Slochower et al [44] for cyclodextrins with GAFF 1.7 and 2.1. Furthermore, progressively adding the dihedral corrections brings the conformational distributions closer to that of GAFF 1.81. The same behavior can be seen in the configuration space of the bound guests, although to a lesser extent (Figs. S6 and S7). The effect is more pronounced for the unbound states of the hosts, which suggests that the dihedral parameter corrections that we introduced, increased the conformational entropy difference between the apo and holo states.

We have further quantified the differences in the conformational entropies by applying Schlitter’s entropy estimator [45] (Table 1). For the GAFF 2.1 force field the conformational entropy difference between the apo and holo states is ${-3.2 \pm 0.2}\,\hbox {kcal}/\hbox {mol}$ in favor of the unbound states at the simulation temperature. Using the sugar-specific dihedrals raises the entropy difference to ${-5.4 \pm 0.7}\,\hbox {kcal}/\hbox {mol}$. Application of the PREMD enhanced sampling, has further increased the entropy difference to ${-10.3 \pm 0.2}\,\hbox {kcal}/\hbox {mol}$ In comparison the value for GAFF 1.81 was ${-13.3 \pm 1.0}\,\hbox {kcal}/\hbox {mol}$.

**Table 1:** Conformational entropies (Schlitter approximation) of the holo and apo states and their effect on the overall binding free energy for each potential used

	Schlitter entropy $(\hbox {J}/(\hbox {mol}\,\hbox{K}))$		T (K)	Contribution to ΔG ${\hbox {(kcal}/\hbox {mol)}}$
	Holo	Apo		
CGenFF 4.1, TIP3P	$392\pm 1$	$439\pm 26$	300.15	$-3\pm 2$
GAFF 1.81, TIP3P	$458\pm 11$	$643\pm 9$	300.15	$-13\pm 1$
GAFF 1.81, OPC3	$465\pm 1$	$641\pm 5$	300.15	$-12.6\pm 0.4$
GAFF 2.11, TIP3P	$364\pm 1$	$417\pm 4$	300.15	$-3.8\pm 0.3$
GAFF 2.11, OPC3	$364\pm 3$	$427\pm 9$	300.15	$-4.5\pm 0.7$
GAFF 2.1, TIP3P	$340\pm 2$	$385\pm 2$	298	$-3.2\pm 0.2$
GAFF 2.1 with sugar-specific dih., TIP3P	$370\pm 2$	$445\pm 9$	298	$-5.4\pm 0.7$
GAFF 2.1 with both dih. sets, TIP3P	$389\pm 2$	$495\pm 11$	298	$-7.6\pm 0.8$
GAFF 2.1 with both dih. sets (PREMD), TIP3P	$391\pm 2$	$535\pm 1$	298	$-10.3\pm 0.2$

The reported values are computed from aggregate trajectories of all host–guest combinations using only the heavy atoms of the unmodified sugars common to all hosts

The conformational entropy provides only an approximation of just one component contributing to the overall binding free energy. Therefore, we should not expect to fully quantitatively explain the changes in binding affinity with these estimates. Nevertheless, it is interesting to note that a consistent trend emerges where modifications to the dihedral potentials increase the conformational entropy difference, primarily by increasing entropy of the apo state. As a result, the entropy reduction upon the guest binding is larger in the GAFF 2.1 variants with the modified dihedrals. This, in part, explains the decrease in the binding affinity upon dihedral adjustments.

## Discussion

The calculated absolute free energies exhibited offsets of varying magnitude depending on the modification of the force field parameters (Fig. 5). However, the inaccuracies in the absolute $\Delta$G values do not necessarily imply that the relative free energy differences $\Delta \Delta$G will be inaccurate as well. We evaluated the prediction accuracies in terms of relative free energies by calculating all non-redundant $\Delta \Delta$G values for each of the guests separately.

**Fig. 7:**
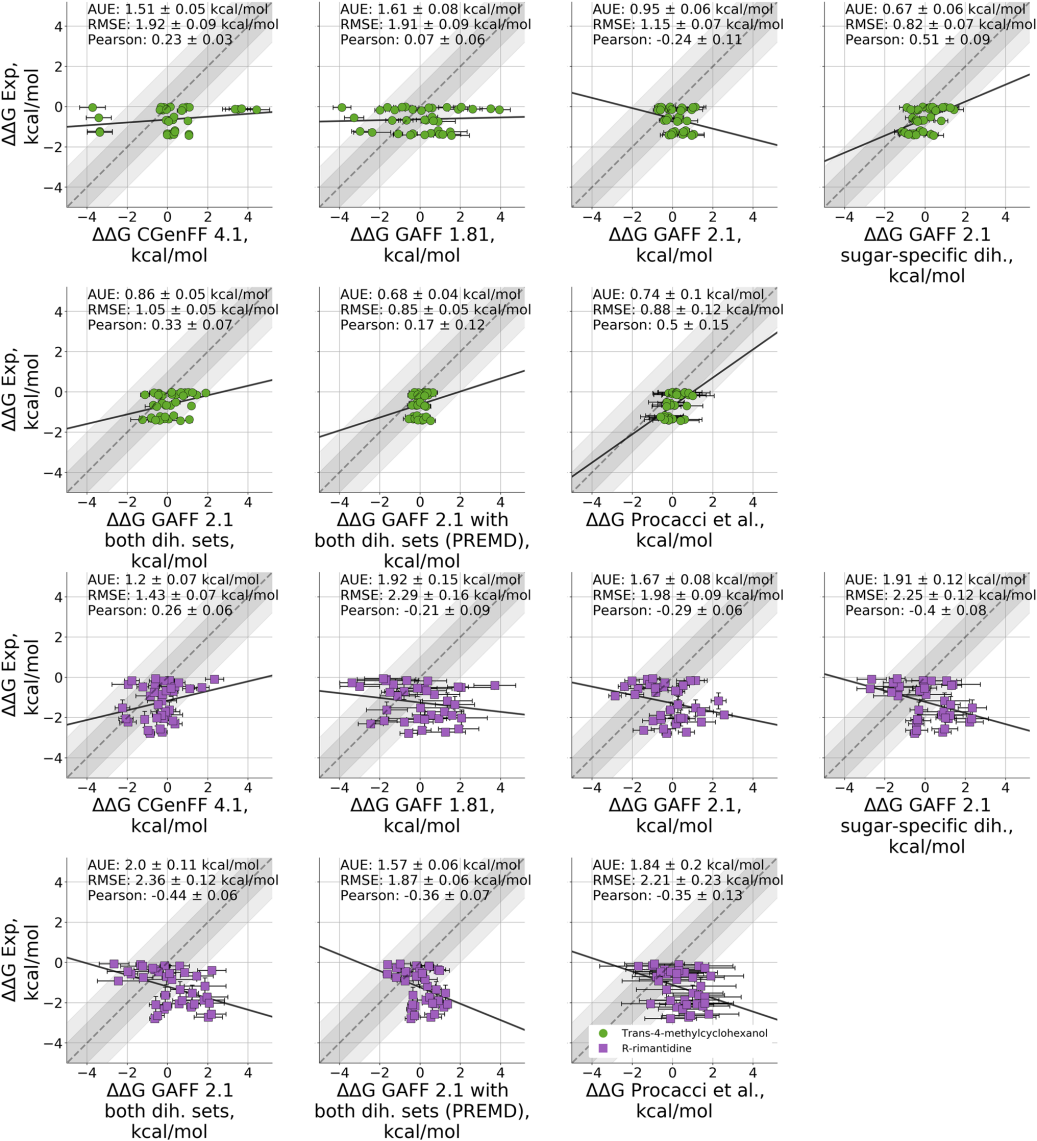
Comparison of relative binding free energies for trans-4-methylcyclohexanol (top row) and R-rimantadine (bottom row) for different versions and modifications of the GAFF force fields

Overall, the extracted relative free energies show either low or no correlation with experiment at all (Fig. 7). In fact, it is difficult to expect to reach high correlations when considering a particularly narrow dynamic range of the $\Delta \Delta$G values calculated from the experimental measurements: up to ${1.5}\,\hbox {kcal}/\hbox {mol}$ for rimantadine and ${2.5}\,\hbox {kcal}/\hbox {mol}$ for methylcyclohexanol (Fig. S8). Given that the state-of-the-art accuracy reached for relative protein-ligand binding affinity calculations is on average $\sim {1}\,\hbox {kcal}/\hbox {mol}$ [18, 46, 47], it is expected that absolute free energy calculations requiring larger perturbations would show a similar or larger deviation from experiment.

The dihedral correction in GAFF 2.1 brings the relative free energies of methylcyclohexanol to the state-of-the-art accuracy of average unsigned error lower than ${1}\,\hbox {kcal}/\hbox {mol}$ (Fig. 7 top). In terms of $\Delta \Delta$G, for this guest GAFF 2.1 is more accurate than GAFF 1.8 and CGenFF 4.1. Applying the dihedral correction allows GAFF 2.1 to outperform all the other considered cases both in terms of the absolute agreement with experiment, as well as in terms of correlation.

For R-rimantadine (Fig. 7 bottom) all the GAFF variants perform poorly in terms of $\Delta \Delta$G, consistently showing negative correlation with the experiment. The highest accuracy for this guest was instead obtained with the CGenFF 4.1 force field, producing an AUE of ${1.2 \pm 0.1}\,\hbox {kcal}/\hbox {mol}$.

The observations in Fig. 7 showcase how the perceived (in)accuracies of the absolute and relative free energy estimates might be deceiving. While Fig. 5 showed a poor accuracy of all the GAFF 2 variants probed in this work, it appears that from the relative affinity point of view the force field was able to capture correct trends for the methylcyclohexanol. Contrarily, even though the absolute $\Delta$G estimates appeared accurate for rimantadine in the case of GAFF 1.8 and Procacci et al. estimations, from the perspective of relative affinities these predictions were of poor quality. Overall, the errors identified in the GAFF 2 over-definition of several sets of dihedrals appear to have an effect on both the absolute $\Delta$G, as well as for the relative free energies (as highlighted by the methylcyclohexanol case).

Another issue that was uncovered in the SAMPL7 challenge was a disparate interpretation of the same force field by different software packages, namely, antechamber 19.0 (also 17.3) and PrimaDORAC 1.0. Firstly, the overdefined dihedrals in GAFF 2 were treated differently: this ambiguity should be resolved by the corrections in the force field. However, other differences in atom type assignment and the subsequent non-bonded and bonded parameter generation remain.

It is known that there are differences in free energy estimates obtained from various software packages [7, 48, 49]. The results may differ due to the particular details of the alchemical calculation implementation [49], or even a choice of the Coulomb’s constant in the molecular dynamics engine [48]. We now highlight one more potential source of such discrepancies, namely the different interpretation of the force field description by the software preparing input for the MD engine. This finding indicates that when comparing outcome from various MD softwares, it is necessary to ensure that the employed force field is interpreted consistently with the force field’s definition.

In this particular case, an analysis of the differences in such interpretations has revealed that increasing the force constants acting on the sugar dihedrals in GAFF 2.1 also increases the accuracy of the predicted binding free energies, suggesting a route for future improvement of GAFF 2.x force fields.

## Conclusions

All in all, participation in the SAMPL7 challenge showed that the non-equilibrium alchemical methods are applicable in free energy calculations of host–guest binding. The consensus force field approach performed as well as the individual best performing force field. A problematic over-definition of the dihedrals in the GAFF 2 force field has been identified. The relative binding energies that were generated from the absolute calculation results showed a good correlation with experiment for the neutral guest, again confirming that overbinding effects were due to a consistent artifact and not an inherent failing of the approach or theory. Finally, we note the discrepancies that may arise between molecular dynamics packages due to the different interpretation of force field parameters.

## Electronic supplementary material

Below is the link to the electronic supplementary material.

Electronic supplementary material 1 (PDF 6106 kb)

## References

[1] Skillman AG (2012). SAMPL3: blinded prediction of host–guest binding affinities, hydration free energies, and trypsin inhibitors. J Comput-Aided Mol Des.

[2] Mobley DL, Liu S, Lim NM, Wymer KL, Perryman AL, Forli S, Deng N, Su J, Branson K, Olson AJ (2014). Blind prediction of HIV integrase binding from the SAMPL4 challenge. J Comput-Aided Mol Des.

[3] Mobley DL, Wymer KL, Lim NM, Guthrie JP (2014). Blind prediction of solvation free energies from the SAMPL4 challenge. J Comput-Aided Mol Des.

[4] Yin J, Henriksen NM, Slochower DR, Shirts MR, Chiu MW, Mobley DL, Gilson MK (2017). Overview of the SAMPL5 host–guest challenge: are we doing better?. J Comput-Aided Mol Des.

[5] Rizzi A, Murkli S, McNeill JN, Yao W, Sullivan M, Gilson MK, Chiu MW, Isaacs L, Gibb BC, Mobley DL (2018). Overview of the SAMPL6 host–guest binding affinity prediction challenge. J Comput-Aided Mol Des.

[6] Işık M, Bergazin TD, Fox T, Rizzi A, Chodera JD, Mobley DL (2020) Assessing the accuracy of octanol–water partition coefficient predictions in the SAMPL6 Part II log P Challenge. J Comput-Aided Mol Des 1–36 10.1007/s10822-020-00295-0 PMC7138020 32107702

[7] Rizzi A, Jensen T, Slochower DR, Aldeghi M, Gapsys V, Ntekoumes D, Bosisio S, Papadourakis M, Henriksen NM, de Groot BL, Cournia Z, Dickson A, Michel J, Gilson MK, Shirts MR, Mobley DL, Chodera JD (2020). The SAMPL6 SAMPLing challenge: assessing the reliability and efficiency of binding free energy calculations. J Comput-Aided Mol Des.

[8] Elisée E, Gapsys V, Mele N, Chaput L, Selwa E, de Groot BL, Iorga BI (2019). Performance evaluation of molecular docking and free energy calculations protocols using the D3R Grand Challenge 4 dataset.. J Comput-Aided Mol Des.

[9] Procacci P, Guarnieri G (2019) SAMPL6 blind predictions of water-octanol partition coefficients using nonequilibrium alchemical approaches. J Comput-Aided Mol Des 1–14 10.1007/s10822-019-00233-9 31624982

[10] Procacci P, Guarrasi M, Guarnieri G (2018). SAMPL6 host-guest blind predictions using a non equilibrium alchemical approach. J Comput-Aided Mol Des.

[11] Wang J, Wolf RM, Caldwell JW, Kollman PA, Case DA (2004). Development and testing of a general amber force field. J Computl Chem.

[12] Vanommeslaeghe K, Hatcher E, Acharya C, Kundu S, Zhong S, Shim J, Darian E, Guvench O, Lopes P, Vorobyov I, Mackerell AD (2010). CHARMM general force field: a force field for drug-like molecules compatible with the CHARMM all-atom additive biological force fields. J Comput Chem.

[13] Wang J, Wang W, Kollman PA, Case DA (2006). Automatic atom type and bond type perception in molecular mechanical calculations. J Mol Graphics Modell.

[14] Wenbo Yu, He X, Vanommeslaeghe K, MacKerell AD (2012). Extension of the CHARMM general force field to sulfonyl-containing compounds and its utility in biomolecular simulations. J Comput Chem.

[15] Aldeghi M, Gapsys V, de Groot BL (2018). Accurate estimation of ligand binding affinity changes upon protein mutation. ACS Cent Sci.

[16] Gapsys V, Michielssens S, Seeliger D, de Groot BL (2016). Accurate and rigorous prediction of the changes in protein free energies in a large-scale mutation scan. Angew Chem Int Ed.

[17] Gapsys V, de Groot BL (2017). Alchemical free energy calculations for nucleotide mutations in protein–DNA complexes. J Chem Theory Comput.

[18] Gapsys V, Pérez-Benito L, Aldeghi M, Seeliger D, van Vlijmen H, Tresadern G, de Groot BL (2020). Large scale relative protein ligand binding affinities using non-equilibrium alchemy. Chem Sci.

[19] Jakalian A, Bush BL, Jack DB, Bayly CI (2000). Fast, efficient generation of high-quality atomic charges. AM1-BCC model: I. Method. J Comput Chem.

[20] Sousa da Silva AW, Vranken WF (2012). ACPYPE—AnteChamber PYthon Parser interfacE. BMC Res Notes.

[21] Vanommeslaeghe K, Prabhu Raman E, MacKerell AD (2012). Automation of the CHARMM General Force Field (CGenFF) II: assignment of bonded parameters and partial atomic charges. J Chem Inf Model.

[22] Kellett K, Kantonen SA, Duggan BM, Gilson MK (2018). Toward expanded diversity of host–guest interactions via synthesis and characterization of cyclodextrin derivatives. J Solut Chem.

[23] Carrazana J, Jover A, Meijide F, Soto VH, Vázquez Tato J (2005). Complexation of Adamantyl compounds by $$\beta $$-Cyclodextrin and Monoaminoderivative. J Phys Chem B.

[24] Mobley DL, Chodera JD, Dill KA (2006). On the use of orientational restraints and symmetry corrections in alchemical free energy calculations. J Chem Phys.

[25] Gapsys V, Michielssens S, Peters JH, de Groot B. L., Leonov H (2015) Calculation of binding free energies. Mol Model Proteins 173–209 10.1007/978-1-4939-1465-4_9 25330964

[26] Jorgensen WL, Chandrasekhar J, Madura JD, Impey RW, Klein ML (1983). Comparison of simple potential functions for simulating liquid water. J Chem Phys.

[27] Joung IS, Cheatham TE (2008). Determination of alkali and halide monovalent ion parameters for use in explicitly solvated biomolecular simulations. J Phys Chem B.

[28] Roux B (1996). Valence selectivity of the gramicidin channel: a molecular dynamics free energy perturbation study. Biophys J.

[29] Venable RM, Luo Y, Gawrisch K, Roux B, Pastor RW (2013). Simulations of anionic lipid membranes: development of interaction-specific ion parameters and validation using NMR data. J Phys Chem B.

[30] Abraham MJ, Murtola T, Schulz R, Páll S, Smith JC, Hess B, Lindahl E (2015). GROMACS: high performance molecular simulations through multi-level parallelism from laptops to supercomputers. SoftwareX.

[31] Darden T, York D, Pedersen L (1993). Particle mesh Ewald: an Nlog(N) method for Ewald sums in large systems. J Chem Phys.

[32] Essmann U, Perera L, Berkowitz ML, Darden T, Lee H, Pedersen LG (1995). A smooth particle mesh Ewald method. J Chem Phys.

[33] Boresch S, Tettinger F, Leitgeb M, Karplus M (2003). Absolute binding free energies: a quantitative approach for their calculation. J Phys Chem B.

[34] Parrinello M, Rahman A (1980). Crystal structure and pair potentials: a molecular-dynamics study. Phys Rev Lett.

[35] Parrinello M, Rahman A (1981). Polymorphic transitions in single crystals: a new molecular dynamics method. J Appl Phys.

[36] Shirts MR, Bair E, Hooker G, Pande VS (2003). Equilibrium free energies from nonequilibrium measurements using maximum-likelihood methods. Phys Rev Lett.

[37] Crooks GE (1999). Entropy production fluctuation theorem and the nonequilibrium work relation for free energy differences. Phys Rev E.

[38] Gapsys V, Michielssens S, Seeliger D, de Groot BL (2015). Pmx: automated protein structure and topology generation for alchemical perturbations. J Comput Chem.

[39] Izadi S, Onufriev AV (2016). Accuracy limit of rigid 3-point water models. J Chem Phys.

[40] Procacci P (2016). Hybrid MPI/openMP implementation of the ORAC molecular dynamics program for generalized ensemble and fast switching alchemical simulations. J Chem Inf Model.

[41] Procacci P (2017). PrimaDORAC: a free web interface for the assignment of partial charges, chemical topology, and bonded parameters in organic or drug molecules. J Chem Inf Model.

[42] Cheng X, Cui G, Hornak V, Simmerling C (2005). Modified replica exchange simulation methods for local structure refinement. J Phys Chem B.

[43] Liu P, Kim B, Friesner RA, Berne BJ (2005). Replica exchange with solute tempering: a method for sampling biological systems in explicit water. PNAS.

[44] Slochower DR, Henriksen NM, Wang L-P, Chodera JD, Mobley DL, Gilson MK (2019). Binding thermodynamics of Host–Guest Systems with SMIRNOFF99Frosst 1.0.5 from the open force field initiative. J Chem Theory Comput.

[45] Schlitter J (1993). Estimation of absolute and relative entropies of macromolecules using the covariance matrix. Chem Phys Lett.

[46] Wang L, Wu Y, Deng Y, Kim B, Pierce L, Krilov G, Lupyan D, Robinson S, Dahlgren MK, Greenwood J (2015). Accurate and reliable prediction of relative ligand binding potency in prospective drug discovery by way of a modern free-energy calculation protocol and force field. J Am Chem Soc.

[47] Kuhn M, Firth-Clark S, Tosco P, Mey ASJS, Mackey M, Michel J (2020). Assessment of binding affinity via alchemical free-energy calculations. J Chem Inf Model.

[48] Shirts MR, Klein C, Swails JM, Yin J, Gilson MK, Mobley DL, Case DA, Zhong ED (2016) Lessons learned from comparing molecular dynamics engines on the SAMPL5 dataset. bioRxiv 077248 10.1007/s10822-016-9977-1 PMC5581938 27787702

[49] Loeffler HH, Bosisio S, Duarte Ramos Matos G, Suh D, Roux B, Mobley DL, Michel J (2018). Reproducibility of free energy calculations across different molecular simulation software packages. J Chem Theory Comput.

