# Supplementary material for: Non-equilibrium approach for binding free energies in cyclodextrins in SAMPL7: force fields and software
Source: J Comput Aided Mol Des. 2020 Nov 24;35(1):49–61. doi: 10.1007/s10822-020-00359-1 (PMC7862541; doi:10.1007/s10822-020-00359-1)
Supplement: Supplementary file 1 — Electronic supplementary material 1 (PDF 6106 kb) [file 10822_2020_359_MOESM1_ESM.pdf]

Supporting Information: Non-equilibrium Approach for Binding Free Energies in Cyclodextrins in SAMPL7: Force Fields and Software

Yuriy Khalak[1], Gary Tresadern[2], Bert L. de Groot[1], and Vytautas Gapsys[1*]

[1]Computational Biomolecular Dynamics Group, Department of Theoretical and Computational Biophysics, Max Planck Institute for Biophysical Chemistry, D-37077, Göttingen, Germany
[2]Computational Chemistry, Janssen Research & Development, Janssen Pharmaceutica N. V., Turnhoutseweg 30, B-2340 Beerse, Belgium
[*]vgapsys@gwdg.de

Below are the excerpts from GAFF force field version 2.11 in Amber18 (gaff2.dat file) illustrating the cases where identical atom types have overdefined dihedral parameters. The line numbers in brackets are for the gaff 2.1 as found in the gaff2.dat of Amber16.

Case 1:

```
LINES 7031-7032 (gaff2.1 6224-6225):
c3-os-c3-c3 1 0.240 0.000 -3 p29 GA AUE=0.4256 RMSE=0.5201 TorType=2
c3-os-c3-c3 1 0.160 0.000 2 p29 GA AUE=0.4256 RMSE=0.5201 TorType=2
...
LINES 7410-7412 (gaff2.1 6603-6605):
c3-c3-os-c3 1 0.910 0.000 -3 lactose1,ccoc GA AUE=1.5236 RMSE=2.4206 TorType=3
c3-c3-os-c3 1 1.000 0.000 -2
c3-c3-os-c3 1 0.000 0.000 1
```

Case 2:

```
LINES 7358 (gaff2.1 6551):
o -c -c3-c3 1 0.270 180.000 2 p14 SS AUE=0.2361 RMSE=0.3321 TorType=3
...
LINES 7407 (gaff2.1 6600-6602):
c3-c3-c -o 1 0.030 180.000 -2 sialic1,t37,t41 GA AUE=0.7374 RMSE=i0.9897 TorType=3
c3-c3-c -o 1 0.550 180.000 -3
c3-c3-c -o 1 0.740 0.000 1
```

Case 3:

```
LINES 7040-7042 (gaff2.1 6233-6235):
c -n -c3-c3 1 0.100 180.000 -4 p19 GA AUE=0.2882 RMSE=0.4031 TorType=2
c -n -c3-c3 1 0.170 0.000 -3 p19 GA AUE=0.2882 RMSE=0.4031 TorType=2
c -n -c3-c3 1 1.020 180.000 1 p19 GA AUE=0.2882 RMSE=0.4031 TorType=2
...
LINES 7119-7121 (gaff2.1 6312-6314):
c3-c3-n -c 1 0.650 180.000 -4 sialic2 GA AUE=1.1541 RMSE=1.2847 TorType=3
c3-c3-n -c 1 0.030 180.000 -3 sialic2 GA AUE=1.1541 RMSE=1.2847 TorType=3
c3-c3-n -c 1 2.260 0.000 1 sialic2 GA AUE=1.1541 RMSE=1.2847 TorType=3
```

Case 4:

```
LINE 7051 (gaff2.1 6244):
hc-c3-c3-c3 1 0.080 0.000 3 m2 SS AUE=0.2468 RMSE=0.2989 TorType=2
...
LINE 7117 (gaff2.1 6310):
c3-c3-c3-hc 1 0.080 0.000 3 t2 SS AUE=0.2507 RMSE=0.3027 TorType=3
```

Case 5:

```
LINE 7088 (gaff2.1 6281):
c2-ce-ca-ca 1 0.505 180.000 2 add6f SS AUE=0.2273 RMSE=0.3302 TorType=3
...
LINE 7248 (gaff2.1 6441):
ca-ca-ce-c2 1 0.618 180.000 2 c24 SS AUE=0.2364 RMSE=0.3330 TorType=3
```

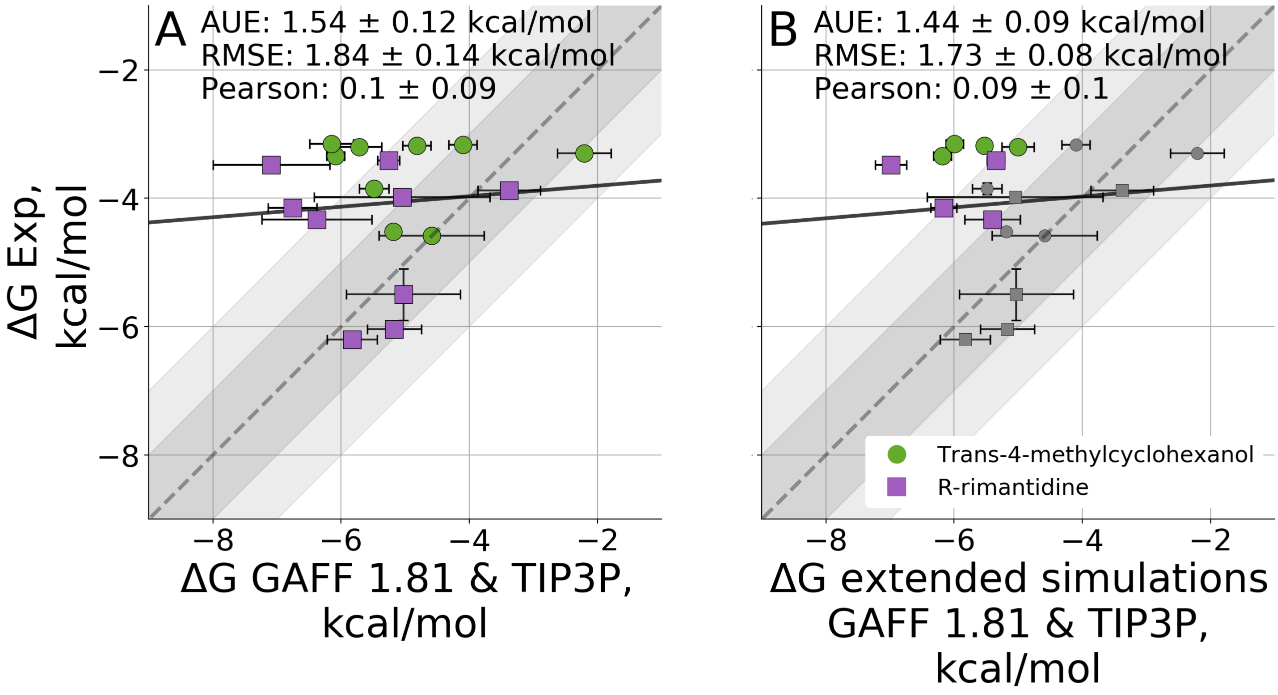

Figure S1: Extended simulations in the GAFF 1.81 forcefield do not reduce overbinding for the outliers (colored).

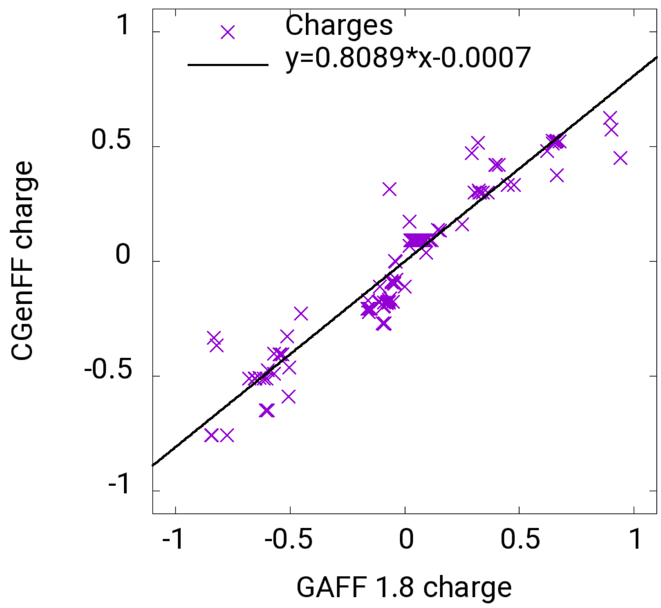

Figure S2: Comparison of sidechain charges in GAFF 1.81 and CGenFF 4.1 for hosts MGLab 19, 23, 24, and 36. These are the hosts with the largest disagreements with experimental ΔG values in the GAFF 1.81 force field. The linear fit illustrates that scaling the GAFF 1.81 charges by a factor of 0.81 approximates CGenFF 4.1 charges.

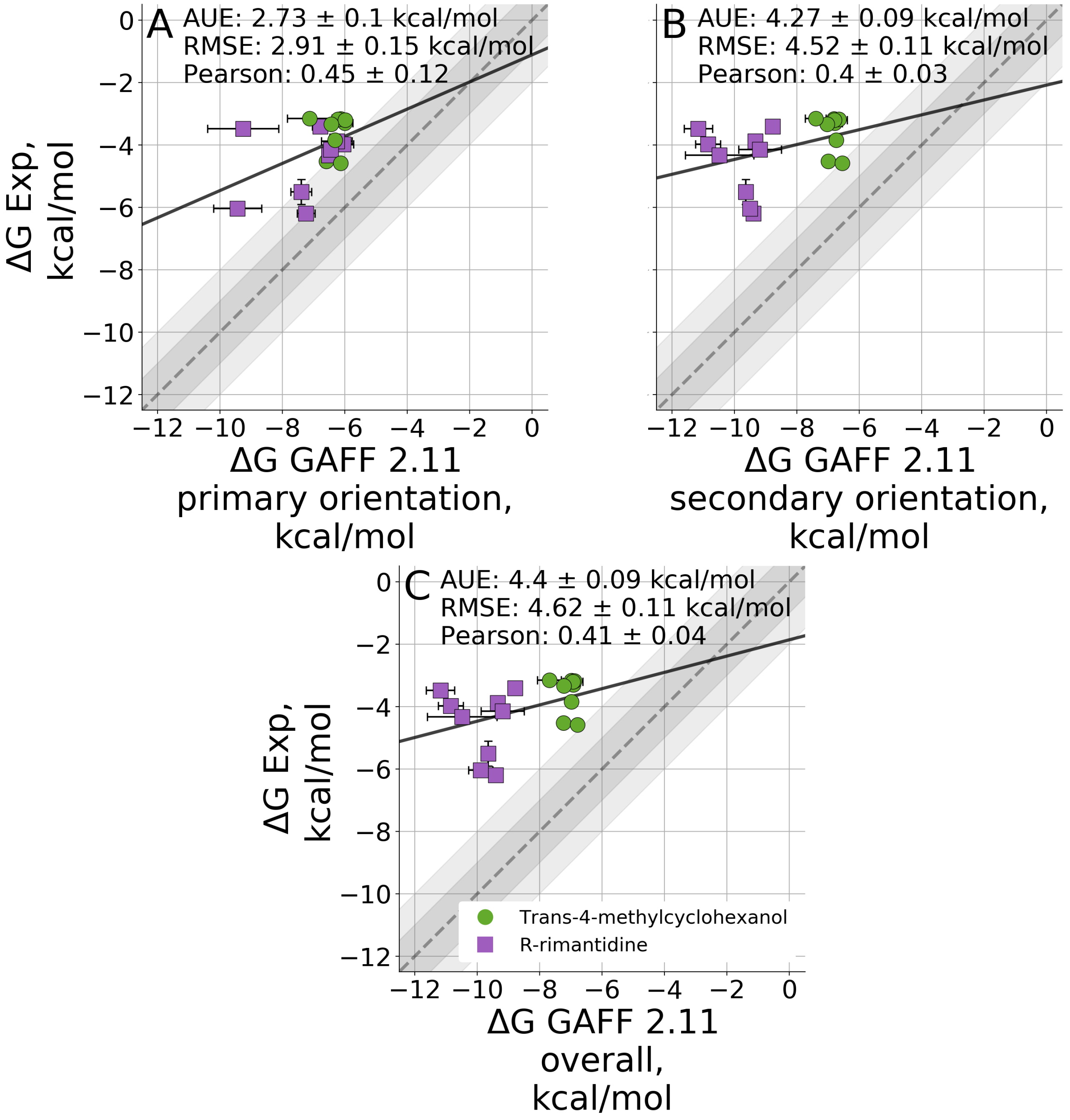

Figure S3: Differences in the binding free energies when binding in the (A) primary and (B) secondary orientations, as well as their Boltzmann weighted average (C) using the GAFF 2.11 forcefield and the TIP3P water model.

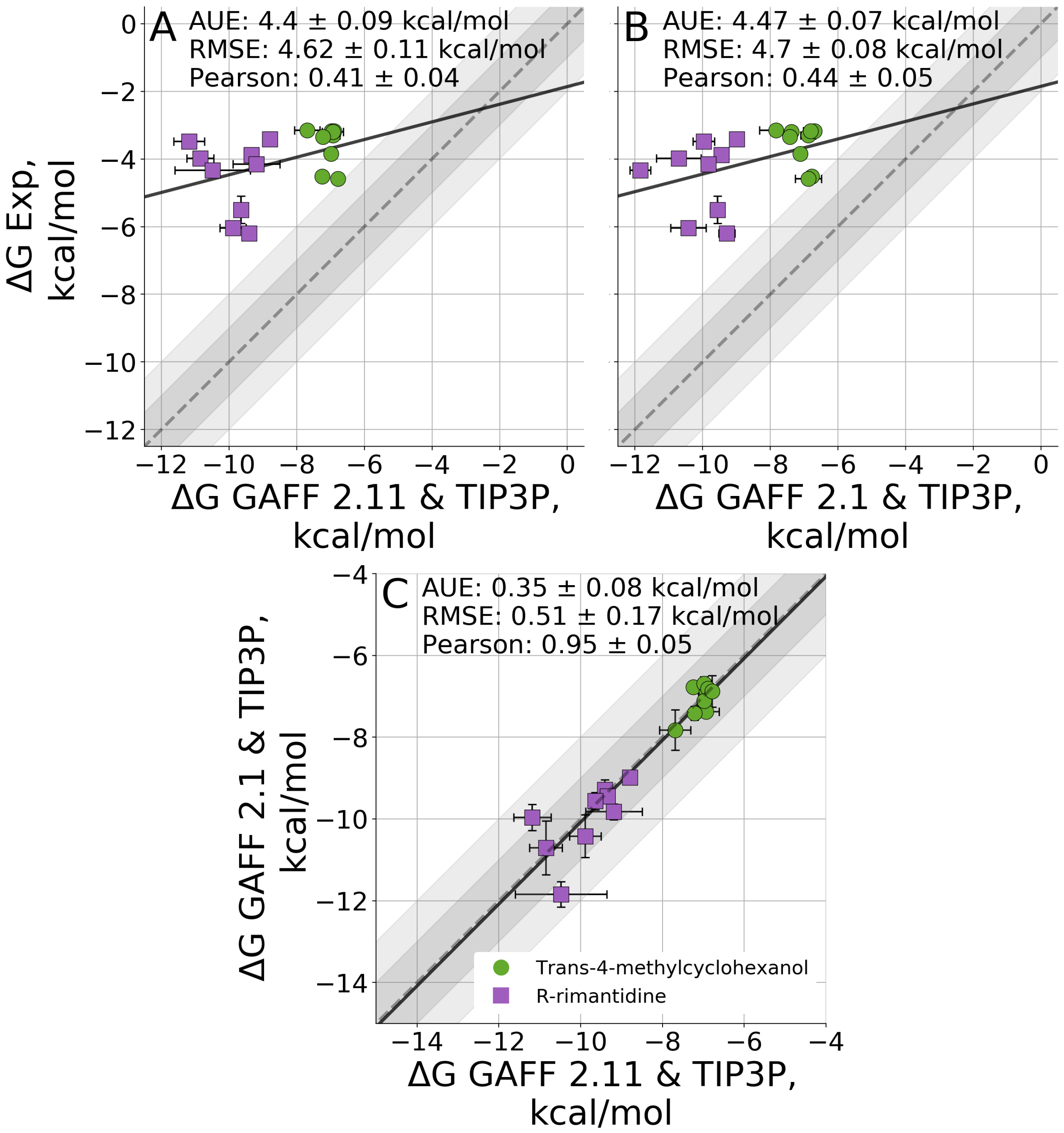

Figure S4: Comparison of the calculation results with the GAFF 2.11 and GAFF 2.1 force fields to the experimentally measured values (A and B, respectively) and to each other (C). The generated topologies differ only in the values of the bond and angle force constants.

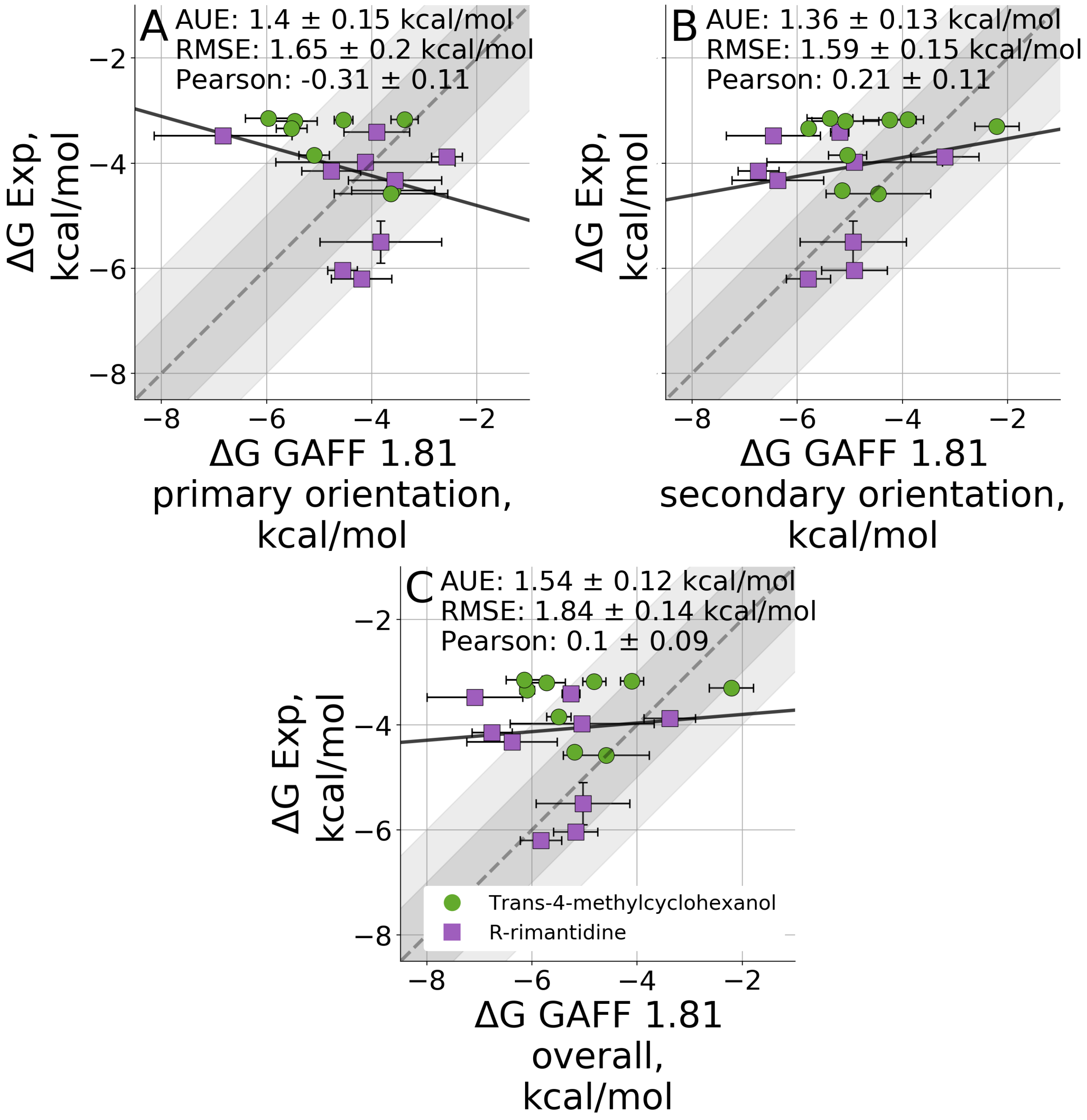

Figure S5: Comparison of the calculation results with the GAFF 1.81 force field for the primary (A) and secondary (B) orientations as well as the overall Boltzmann averaged estimates (C) with the experimentallly measured values.

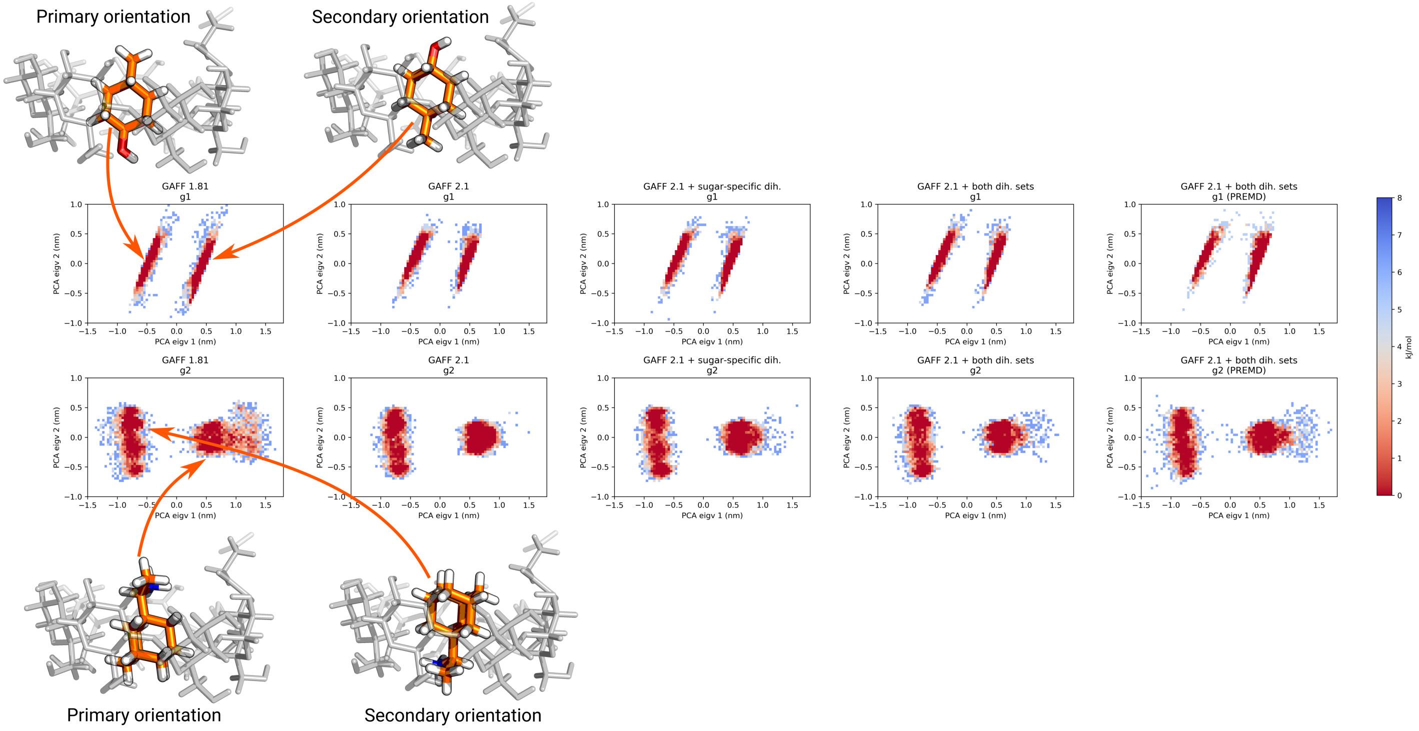

Figure S6: Free energy surfaces of guest conformations obtained with different potentials projected on non-mass-wheighted heavy atom primary components from GAFF 1.81 trajectories. All simulations depicted used TIP3P. Both trans-4-methylcyclohexanol (top) and R-rimantadine (bottom) can be cleary separated into up and down states, but their distributions do not change much between different potentials. The eigenvector with the largest principal component separates two binding orientations (primary and secondary) which are identified as distinct clusters in the projections.

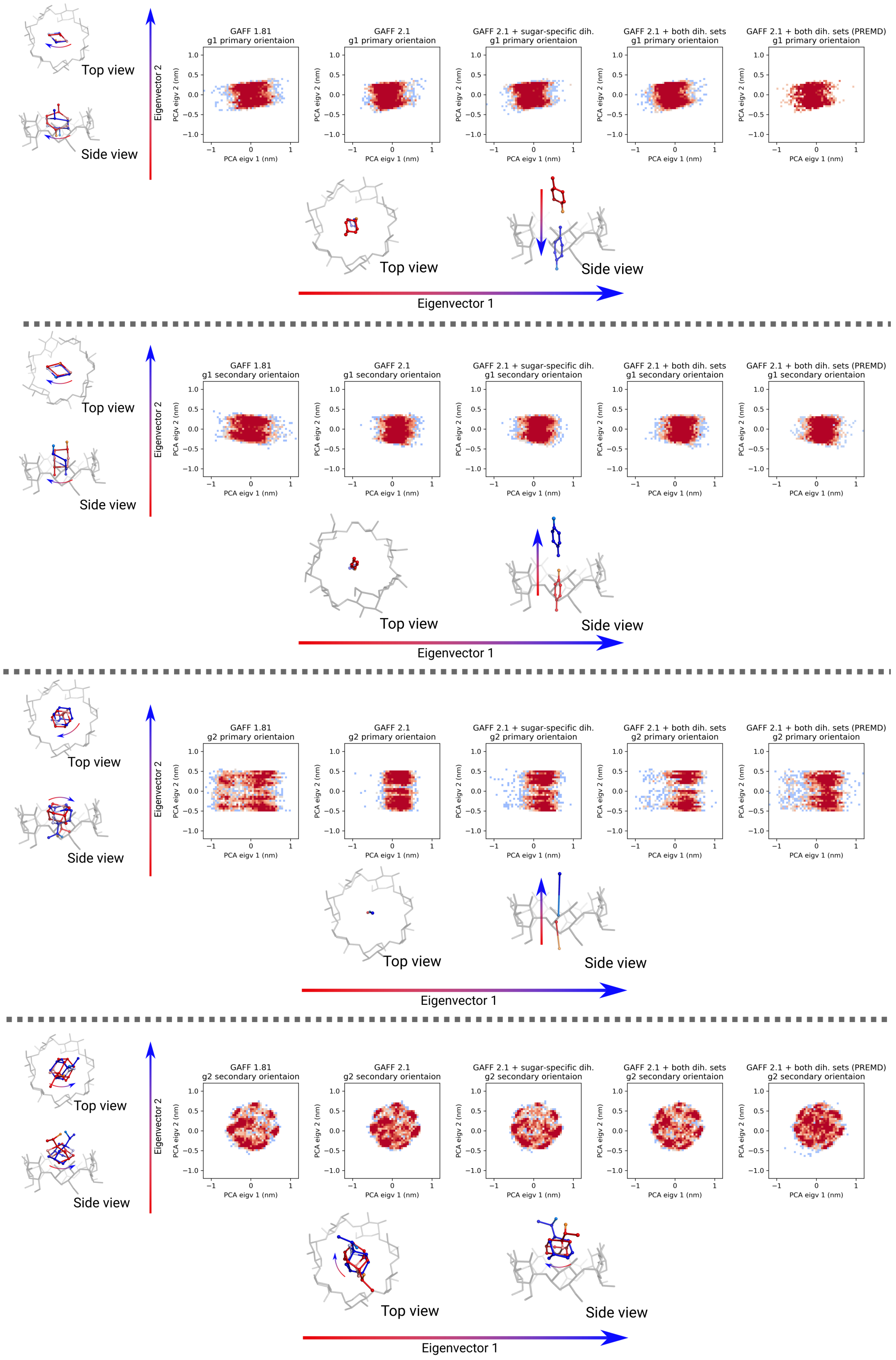

Figure S7: Free energy surfaces of guest conformations obtained with different potentials projected on non-mass-wheighted heavy atom primary components from GAFF 1.81 trajectories separately for each guest and orientation. Modifying the dihedral potential for GAFF 2.1 enables guests to bind in a larger range of conformations, bringing the binding distributions closer to those of GAFF 1.81. Structural representations depict interpolations along the two principal components with the largest eigenvalues.

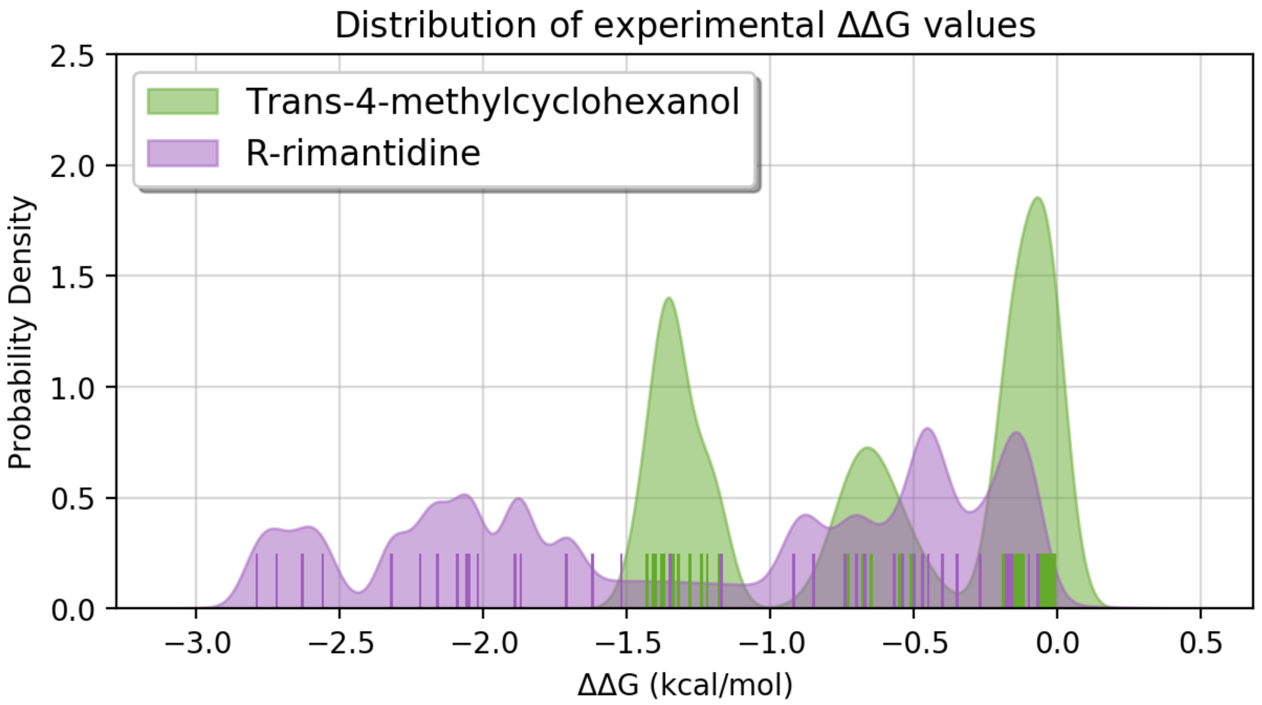

Figure S8: Experimental distributions of relative binding free energies for both guests. The pale regions represent the overall distributions assuming Gaussian error distributions in each absolute free energy measurement. Vertical lines indicate each individual relative binding free energy.
